# Reversible dilated cardiomyopathy as a complication of adrenal cortex insufficiency: a case report

**DOI:** 10.1186/s13256-018-1899-1

**Published:** 2018-11-21

**Authors:** Mohammad Alkhateeb, Mohammad Alsakkal, Mohammad Nour Alfauri, Diana Alasmar

**Affiliations:** 1 Damascus University - Faculty of Medicine, Damascus, Syria; 2 Pediatric Hospital in Damascus, Damascus, Syria

**Keywords:** Primary adrenal cortex insufficiency, Dilated cardiomyopathy, Glucocorticoids

## Abstract

**Background:**

Cardiovascular manifestations associated with Addison’s disease are previously documented. We described a case of an 11-year-old girl who developed dilated cardiomyopathy as a complication to Addison’s disease. Glucocorticoid replacement therapy resulted in near-complete recovery of cardiac function. It is the first reported case of reversible cardiomyopathy as a complication of primary adrenal insufficiency in Syria.

**Case presentation:**

An 11-year-old Caucasian girl with no significant past medical history presented with abdominal pain, vomiting after meals, and a low-grade fever. A physical examination and laboratory evaluation suggested primary adrenal insufficiency. An echocardiogram showed changes consistent with dilated cardiomyopathy. Causes of primary adrenal insufficiency other than autoimmune were excluded.

**Conclusions:**

Dilated cardiomyopathy is a rare complication of primary adrenal insufficiency. Proper treatment of adrenal insufficiency with glucocorticoid replacement therapy resulted in restoration of normal cardiac function.

## Background

Addison’s disease, also known as primary adrenal insufficiency, is a disorder that occurs when the body produces insufficient amounts of cortisol and aldosterone. The failure of adrenal glands is most commonly the result of autoimmune disease. Other causes include tuberculosis, cancer or its treatment, and bleeding into the adrenal glands [1]. It is associated with nonspecific symptoms such as fatigue, weight loss, skin hyperpigmentation, hypoglycemia, and nausea. Cardiovascular manifestations of Addison’s disease include hypotension, arrhythmias, and syncope [2].

In extremely rare instances, Addison’s disease can be complicated with dilated cardiomyopathy. There are only a handful of such cases in the literature (see later). Here we describe a case of Addison’s disease associated with dilated cardiomyopathy which responded to treatment with corticosteroids.

## Case presentation

An 11-year-old Caucasian girl presented to the Pediatric Hospital in Damascus with a 2-month history of diffuse abdominal pain, yellowish vomiting after meals, a low-grade fever (38.5°) that responded to anti-pyretic medications, malaise, and polyuria. A physical examination on admission revealed mild pallor, light pigmentation on the lips, and a body mass index (BMI) of 17.9 kg/m^2^. Her vital signs were as following: blood pressure 80/50 mmHg, temperature 37.0°, heart rate (HR) 100/minute, and respiratory rate (RR) 20/minute. There was no jugular venous distention, no lymphadenopathies and no organomegalies. Heart and lung auscultation were normal. She had no signs of peripheral edema. Her Mini Mental State Examination score was 26/30 and her Glasgow Coma Scale was 15/15. Her muscle strength, tone, and reflexes were all normal. Sensory examination and cranial nerves were normal. She had been treated with nitrofurantoin for recurrent urinary tract infections. Her medical history included no other medications. There was no significant family, social, or environmental history. Her Caucasian parents were not related.

A complete blood count (CBC) showed reduced white blood cells (WBC) of 3940 cells/mm^3^ with neutrophils/lymphocytes (N/L) of 52/26, hemoglobin (HB) 9 g/dL, platelets (PLT) 240,000/mm^3^, and mean corpuscular volume (MCV) 77 fL. Laboratory studies showed: sodium 129 mEq/L, potassium 4.53 mEq/L, creatinine 0.97 mg/dL, chloride 105 mEq/L, ionized calcium 1.25 mmol/L, alkaline phosphatase (ALP) 223 IU/L, fasting glucose 97 mg/dL, glycated hemoglobin (HBA1c) 4%, C-reactive protein (CRP) 19.6 mg/L, and erythrocyte sedimentation rate (ESR) 112 mm/hour. Thyroid-stimulating hormone (TSH) and free thyroxine (FT4) were within normal range. Serial measurements of serum glucose were within normal limits. A blood smear showed hypochromic microcytic anemia. Widal, Wright, and tuberculin tests were negative.

**Fig. 1:**
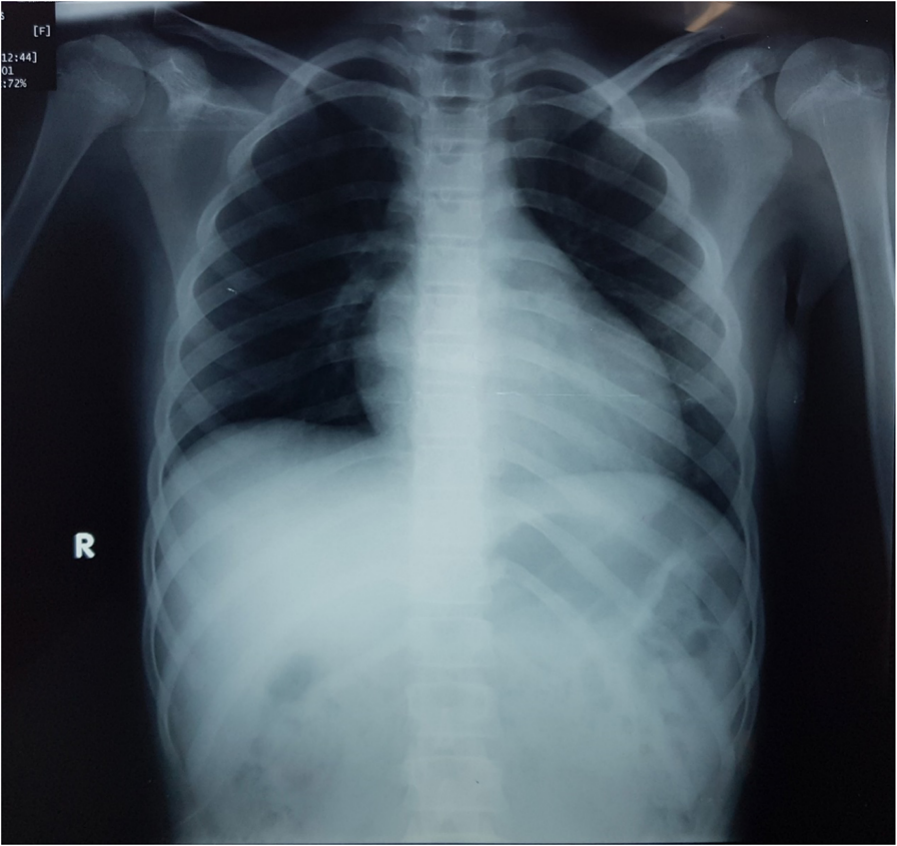
Posteroanterior chest-X ray showing increased cardiopulmonary index

Abdominal and pelvic ultrasonography was normal. An upper gastrointestinal endoscopy revealed erosions in the fundus and body of the stomach. A chest X-ray showed increased cardiothoracic ratio (Fig. 1). An echocardiogram indicated dilated left ventricle (left ventricular dimensions were 55 × 44 mm), decreased fractional shortening (Fs; 13%), an ejection fraction (EF) of 26%, paradoxical septal movements, pulmonary blood flow of 0.7 m/second, and grade 2 mitral valve insufficiency. The right chambers were within normal range.

Urine culture revealed growth of *Klebsiella* species. Voiding cystourethrogram revealed no abnormal findings.

Based on her physical examination, she was judged to be euvolemic. To correct the euvolemic hyponatremia, water intake was restricted to 75% of the calculated daily need. Despite this, hyponatremia did not resolve. A cardiac ultrasound suggested dilated cardiomyopathy so cardiomyopathy management protocol (digoxin, furosemide, spironolactone, and captopril) was initiated with no remarkable improvement. She was also started on trimethoprim/sulfamethoxazole for the urinary tract infection until urine culture became negative.

**Fig. 2:**
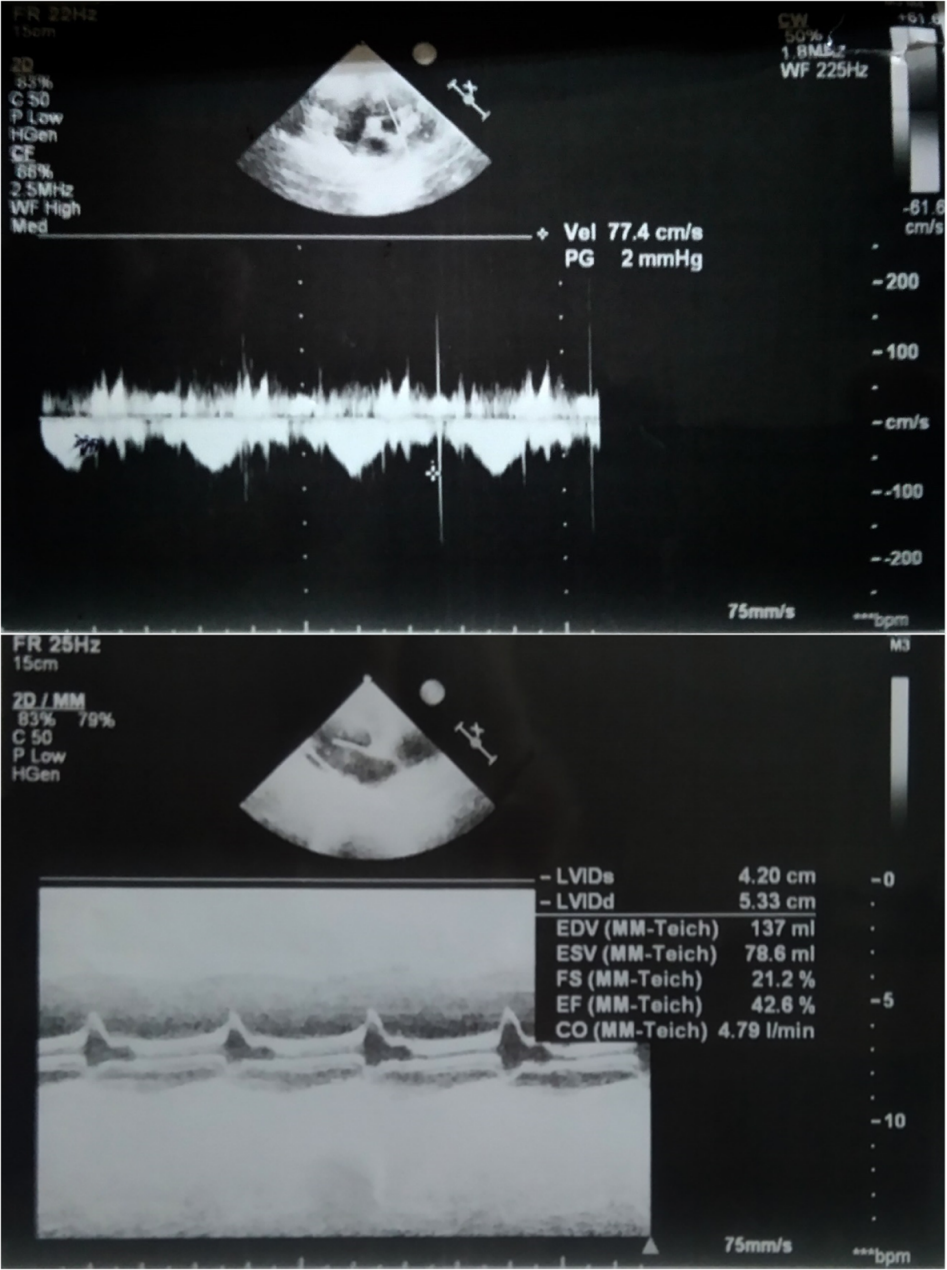
Parasternal long-axis transthoracic echocardiogram 1 week after initiating treatment shows minimal improvement in cardiac function

Her history, along with the physical examination findings and laboratory evaluation suggested adrenal insufficiency. To confirm this, she underwent tests for the adrenal cortex function and the results were as following: random serum cortisol was 4.25 mcg/dL, adrenocorticotropic hormone (ACTH) 1500 pg/ml, and 17-hydroxyprogesterone 0.7 ng/ml. Hyponatremia, low cortisol, and high ACTH along with her symptoms suggested primary adrenal cortex insufficiency. She was treated with 100 mg/m^2^ intravenously administered hydrocortisone which was gradually reduced to 20 mg/m^2^ orally administered hydrocortisone before discharge. Remarkable improvement was noted within days of starting treatment. A heart echocardiogram before discharge showed considerable improvement (dimensions were 53 × 42 mm, EF 42.6%, and Fs 21.2%; Fig. 2). After 2 weeks of hospitalization, she was discharged from our hospital on orally administered 20 mg/m^2^ hydrocortisone.

During the 6 months after discharge, she was followed-up to observe the clinical, laboratory, and radiologic improvements. Serial echocardiograms showed gradual restoration of cardiac function to near-normal status (EF 50% and FS 26%).

There were no signs of chronic mucocutaneous candidiasis or autoimmune hypoparathyroidism which, if present, would suggest autoimmune polyglandular syndrome type 1 (APS1). Since autoimmune polyglandular syndromes, congenital adrenal hyperplasia, bleeding into the adrenals, and tuberculosis were excluded and she had no history of glucocorticoid therapy, the cause of her adrenal insufficiency is mostly autoimmune. Immunologic tests to confirm this were not available.

## Discussion

We present a case of adrenal insufficiency in an 11-year-old girl which was complicated with dilated cardiomyopathy. Glucocorticoid replacement therapy led to near-complete restoration of normal cardiac function.

Primary adrenal insufficiency is rare in children. Among 103 children diagnosed as having primary adrenal insufficiency in a single center in Canada during a 20-year period, congenital adrenal hyperplasia was the underlying cause in 74 patients (71.8%), while autoimmune cases accounted for 12.7% [3].

Dilated cardiomyopathy is an extremely rare complication of adrenal insufficiency. Including our case, we identified seven reports of pediatric adrenal insufficiency accompanied with dilated cardiomyopathy in the literature (see Table 1). The patient was a female in six of the cases (85.7%). Age at presentation varied from several hours to 13 years.

**Table 1:** The previous reports of pediatric adrenal insufficiency cases complicated with dilated cardiomyopathy

Children	Sex	Age (years)	Cause	Timing of cardiomyopathy in relation to adrenal insufficiency treatment
Derish *et al*. (1996) [4]	Male	11	Idiopathic	After
Conwell *et al*. (2003) [5]	Female	13	Autoimmune	After
Ödek *et al.* (2017) [6]	Female	6	Autoimmune	After
Wiltshire *et al.* (2004) [14]	Female	8	Idiopathic	Before
Boston *et al*. (1994) [9]	Female	0	Congenital adrenal hyperplasia	Before
Wani *et al*. (2013) [8]	Female	9	Autoimmune polyglandular syndrome type 1	Before

Despite having a low EF, our patient did not present with edemas, possibly due to the impaired renin-angiotensin-aldosterone system caused by Addison’s disease. Therefore, it is important to note that Addison’s disease could mask the accompanying heart failure, and we suggest performing heart echography for pediatric patients with Addison’s disease if heart dysfunction is suspected by history or physical examination.

There are several reports of cardiomyopathy associated with secondary adrenal insufficiency, which suggest that a hypocortisol state may be the cause of cardiomyopathy regardless of its etiology. However, there are several reports where cardiomyopathy first presented after initiating treatment with corticosteroids [4–7]. In our case, cardiomyopathy preceded the treatment with corticosteroids and improved after it. Reversible dilated cardiomyopathy was also described in APS1 [8], as well as in congenital adrenal hyperplasia [9]. Walker and Butt described a case of heart failure in a 6-year-old boy with adrenoleukodystrophy [10]. Adrenal insufficiency has also been associated with transient left ventricular apical ballooning known as Takotsubo cardiomyopathy. This type occurs exclusively in adult patients [11].

The mechanism of cardiomyopathy with adrenal insufficiency is not fully understood. It is known that glucocorticoid deficiency downregulates the expression of adrenergic receptors resulting in cardiovascular collapse [12]. Rao *et al.* showed that adrenalectomized rat models have diminished Ca^2+^ uptake by the sarcoplasmic reticulum that was greatly reversed by dexamethasone treatment [13]. Decreased Ca^2+^ content in the sarcoplasmic reticulum may underlie the decreased cardiac contractile function [13]. Further research is needed to identify the exact mechanism by which adrenal insufficiency causes cardiomyopathy that is reversible with glucocorticoid replacement therapy.

## Conclusions

The case presented here underscores two important issues: the rare cardiac manifestation of Addison disease in the form of dilated cardiomyopathy, and the reversibility of this cardiomyopathy if the underlying disease is treated. The occurrence of this condition in a young female is also consistent with patients’ characteristics mentioned in earlier reports.

## Abbreviations

ACTH: Adrenocorticotropic hormone
ALP: Alkaline phosphatase
APS1: Autoimmune polyglandular syndrome type 1
BMI: Body mass index
CBC: Complete blood count
CRP: C-reactive protein
EF: Ejection fraction
ESR: Erythrocyte sedimentation rate
Fs: Fractional shortening
FT4: Free thyroxine
HB: Hemoglobin
HBA1c: Glycated hemoglobin
HR: Heart rate
MCV: Mean corpuscular volume
N/L: Neutrophils/lymphocytes
PLT: Platelets
RR: Respiratory rate
TSH: Thyroid-stimulating hormone
WBC: White blood cells

## References

[1] Martorell PM, Roep BO, Smit JWA. Autoimmunity in Addison’s disease. Neth J Med. 2002;60(7):269–75. 12430572

[2] Mozolevska V, Schwartz A, Cheung D, Shaikh B, Bhagirath KM, Jassal DS. Addison’s Disease and Dilated Cardiomyopathy: A Case Report and Review of the Literature. Case Rep Cardiol. 2016;2016:1–5. 10.1155/2016/4362514. PMC5149592 28003914

[3] Perry R, Kecha O, Paquette J, Huot C, Van Vliet G, Deal C. Primary adrenal insufficiency in children: Twenty years experience at the Sainte-Justine Hospital, Montreal. J Clin Endocrinol Metab. 2005;90(6):3243–50. 10.1210/jc.2004-0016. 15811934

[4] Derish M, Eckert K, Chin C. Reversible cardiomyopathy in a child with Addison’s disease. Intensive Care Med. 1996;22(5):460–3. 10.1007/BF01712167. 8796402

[5] Conwell LS, Gray LM, Delbridge RG, Thomsett MJ, Batch JA. Reversible cardiomyopathy in paediatric Addison’s disease--a cautionary tale. J Pediatr Endocrinol Metab. 2003;16(8):1191–5. 10.1515/JPEM.2003.16.8.1191. 14594181

[6] Ödek Ç, Kendirli T, Kocaay P, et al. Acute reversible cardiomyopathy and heart failure in a child with acute adrenal crisis. Paediatr Int Child Health. 2017;37(2):148-51. 10.1080/20469047.2015.1120410. 27077627

[7] Wolff B, Machill K, Schulzki I, Schumacher D, Werner D. Acute reversible cardiomyopathy with cardiogenic shock in a patient with Addisonian crisis: A case report. Int J Cardiol. 2007;116(2):71–3. 10.1016/j.ijcard.2006.07.207. 17079038

[8] Wani AI, Farooqui KJ, Bashir MI, Mir SA, *et al.* Autoimmune polyglandular syndrome type 1 with reversible dilated cardiomyopathy: complete recovery after correction of hypocalcemia and hypocortisolemia. J Pediatr Endocrinol Metab. 2013;26:373–6. 10.1515/jpem-2012-0201. 23327823

[9] Boston BA, DeGroff C, Hanna CE, Reller M. Reversible cardiomyopathy in an infant with unrecognized congenital adrenal hyperplasia. J Pediatr. 1994;124(6):936–8. 10.1016/S0022-3476(05)83186-5. 8201481

[10] Walker C, Butt W. A case of cardiovascular collapse due to adrenal insufficiency. J Paediatr Child Health. 1988;24(3):197–8. 10.1111/j.1440-1754.1988.tb00325.x. 3421879

[11] Shimizu M, Monguchi T, Takano T, Miwa Y. Isolated ACTH deficiency presenting with severe myocardial dysfunction. J Cardiol Cases. 2011;4(1):e26–30. 10.1016/j.jccase.2011.05.004. PMC6265152 30546710

[12] Ullian ME. The role of corticosteriods in the regulation of vascular tone. Cardiovasc Res. 1999;41(1):55–64. 10.1016/S0008-6363(98)00230-2. 10325953

[13] Rao MK, Xu A, Narayanan N. Glucocorticoid modulation of protein phosphorylation and sarcoplasmic reticulum function in rat myocardium. Am J Physiol Heart Circ Physiol. 2001;281(1):H325–33. 10.1152/ajpheart.2001.281.1.H325. 11406500

[14] Wiltshire EJ, Wilson R, Pringle KC. Addison’s disease presenting with an acute abdomen and complicated by cardiomyopathy. J Paediatr Child Health. 2004;40(11):644-5. 10.1111/j.1440-1754.2004.00495.x. 15469537

